# Supplementary material for: A Critical View of the Application of the APEX Software (Aqueous Photochemistry of Environmentally-Occurring Xenobiotics) to Predict Photoreaction Kinetics in Surface Freshwaters
Source: Molecules. 2019 Dec 18;25(1):9. doi: 10.3390/molecules25010009 (PMC7017383; doi:10.3390/molecules25010009)
Supplement: Supplementary file 1 [file molecules-25-00009-s001.zip › 3.2.4_gcc-4.4.0.zip!/3.2.4_gcc-4.4.0/3.2.4_gcc-4.4.0/doc/octave/html/interpreter/Filesystem-Utilities.html]

Filesystem Utilities - Untitled


Next: File Archiving Utilities,
Previous: Timing Utilities,
Up: System Utilities


---

### 34.2 Filesystem Utilities

Octave includes the following functions for renaming and deleting files,
creating, deleting, and reading directories, and for getting information
about the status of files.

— Built-in Function: [err, msg] = **rename** (old, new)  
> Change the name of file old to new.
>
> If successful, err is 0 and msg is an empty string.
> Otherwise, err is nonzero and msg contains a
> system-dependent error message.
>
>
>
> **See also:** ls, dir.

— Built-in Function: [err, msg] = **link** (old, new)  
> Create a new link (also known as a hard link) to an existing file.
>
> If successful, err is 0 and msg is an empty string.
> Otherwise, err is nonzero and msg contains a
> system-dependent error message.
>
>
>
> **See also:** symlink.

— Built-in Function: [err, msg] = **symlink** (old, new)  
> Create a symbolic link new which contains the string old.
>
> If successful, err is 0 and msg is an empty string.
> Otherwise, err is nonzero and msg contains a
> system-dependent error message.
>
>
>
> **See also:** link, readlink.

— Built-in Function: [result, err, msg] = **readlink** (symlink)  
> Read the value of the symbolic link symlink.
>
> If successful, result contains the contents of the symbolic link
> symlink, err is 0 and msg is an empty string.
> Otherwise, err is nonzero and msg contains a
> system-dependent error message.
>
>
>
> **See also:** link, symlink.

— Built-in Function: [err, msg] = **unlink** (file)  
> Delete the file named file.
>
> If successful, err is 0 and msg is an empty string.
> Otherwise, err is nonzero and msg contains a
> system-dependent error message.

— Built-in Function: [files, err, msg] = **readdir** (dir)  
> Return names of the files in the directory dir as a cell array of
> strings. If an error occurs, return an empty cell array in files.
>
> If successful, err is 0 and msg is an empty string.
> Otherwise, err is nonzero and msg contains a
> system-dependent error message.
>
>
>
> **See also:** dir, glob.

— Built-in Function: [status, msg, msgid] = **mkdir** (dir)  
— Built-in Function: [status, msg, msgid] = **mkdir** (parent, dir)  
> Create a directory named dir in the directory parent.
>
> If successful, status is 1, with msg and msgid empty
> character strings. Otherwise, status is 0, msg contains a
> system-dependent error message, and msgid contains a unique
> message identifier.
>
>
>
> **See also:** rmdir.

— Built-in Function: [status, msg, msgid] = **rmdir** (dir)  
— Built-in Function: [status, msg, msgid] = **rmdir** (dir, `"s"`)  
> Remove the directory named dir.
>
> If successful, status is 1, with msg and msgid empty
> character strings. Otherwise, status is 0, msg contains a
> system-dependent error message, and msgid contains a unique
> message identifier.
>
> If the optional second parameter is supplied with value `"s"`,
> recursively remove all subdirectories as well.
>
>
>
> **See also:** mkdir, confirm\_recursive\_rmdir.

— Built-in Function: val = **confirm\_recursive\_rmdir** ()  
— Built-in Function: old\_val = **confirm\_recursive\_rmdir** (new\_val)  
> Query or set the internal variable that controls whether Octave
> will ask for confirmation before recursively removing a directory tree.

— Built-in Function: [err, msg] = **mkfifo** (name, mode)  
> Create a fifo special file named name with file mode mode
>
> If successful, err is 0 and msg is an empty string.
> Otherwise, err is nonzero and msg contains a
> system-dependent error message.

— Built-in Function: **umask** (mask)  
> Set the permission mask for file creation. The parameter mask
> is an integer, interpreted as an octal number. If successful,
> returns the previous value of the mask (as an integer to be
> interpreted as an octal number); otherwise an error message is printed.

— Built-in Function: [info, err, msg] = **stat** (file)  
— Built-in Function: [info, err, msg] = **lstat** (file)  
> Return a structure s containing the following information about
> file.
>
> `dev`: ID of device containing a directory entry for this file. `ino`: File number of the file. `mode`: File mode, as an integer. Use the functions `S_ISREG`, `S_ISDIR`, `S_ISCHR`, `S_ISBLK`, `S_ISFIFO`, `S_ISLNK`, or `S_ISSOCK` to extract information from this value. `modestr`: File mode, as a string of ten letters or dashes as would be returned by `ls -l`. `nlink`: Number of links. `uid`: User ID of file's owner. `gid`: Group ID of file's group. `rdev`: ID of device for block or character special files. `size`: Size in bytes. `atime`: Time of last access in the same form as time values returned from `time`. See Timing Utilities. `mtime`: Time of last modification in the same form as time values returned from `time`. See Timing Utilities. `ctime`: Time of last file status change in the same form as time values returned from `time`. See Timing Utilities. `blksize`: Size of blocks in the file. `blocks`: Number of blocks allocated for file.
>
> If the call is successful err is 0 and msg is an empty
> string. If the file does not exist, or some other error occurs, s
> is an empty matrix, err is −1, and msg contains the
> corresponding system error message.
>
> If file is a symbolic link, `stat` will return information
> about the actual file that is referenced by the link. Use `lstat`
> if you want information about the symbolic link itself.
>
> For example,
>
> ```
>           [s, err, msg] = stat ("/vmlinuz")
>                 => s =
>                   {
>                     atime = 855399756
>                     rdev = 0
>                     ctime = 847219094
>                     uid = 0
>                     size = 389218
>                     blksize = 4096
>                     mtime = 847219094
>                     gid = 6
>                     nlink = 1
>                     blocks = 768
>                     mode = -rw-r--r--
>                     modestr = -rw-r--r--
>                     ino = 9316
>                     dev = 2049
>                   }
>                => err = 0
>                => msg =
> ```

— Built-in Function: [info, err, msg] = **fstat** (fid)  
> Return information about the open file fid. See `stat`
> for a description of the contents of info.

— Function File: [status, msg, msgid] = **fileattrib** (file)  
> Return information about file.
>
> If successful, status is 1, with result containing a
> structure with the following fields:
>
> `Name`: Full name of file. `archive`: True if file is an archive (Windows). `system`: True if file is a system file (Windows). `hidden`: True if file is a hidden file (Windows). `directory`: True if file is a directory. `UserRead` `GroupRead` `OtherRead`: True if the user (group; other users) has read permission for file. `UserWrite` `GroupWrite` `OtherWrite`: True if the user (group; other users) has write permission for file. `UserExecute` `GroupExecute` `OtherExecute`: True if the user (group; other users) has execute permission for file.
>
> If an attribute does not apply (i.e., archive on a Unix system) then
> the field is set to NaN.
>
> With no input arguments, return information about the current
> directory.
>
> If file contains globbing characters, return information about
> all the matching files.
>
>
>
> **See also:** glob.

— Function File: **isdir** (f)  
> Return true if f is a directory.

— Built-in Function: **glob** (pattern)  
> Given an array of strings (as a char array or a cell array) in
> pattern, return a cell array of file names that match any of
> them, or an empty cell array if no patterns match. Tilde expansion
> is performed on each of the patterns before looking for matching file
> names. For example,
>
> ```
>           glob ("/vm*")
>                => "/vmlinuz"
> ```
>
>
>
>
> **See also:** dir, ls, stat, readdir.

— Built-in Function: **fnmatch** (pattern, string)  
> Return 1 or zero for each element of string that matches any of
> the elements of the string array pattern, using the rules of
> filename pattern matching. For example,
>
> ```
>           fnmatch ("a*b", {"ab"; "axyzb"; "xyzab"})
>                => [ 1; 1; 0 ]
> ```

— Built-in Function: **file\_in\_path** (path, file)  
— Built-in Function: **file\_in\_path** (path, file, "all")  
> Return the absolute name of file if it can be found in
> path. The value of path should be a colon-separated list of
> directories in the format described for `path`. If no file
> is found, return an empty matrix. For example,
>
> ```
>           file_in_path (EXEC_PATH, "sh")
>                => "/bin/sh"
> ```
>
> If the second argument is a cell array of strings, search each
> directory of the path for element of the cell array and return
> the first that matches.
>
> If the third optional argument `"all"` is supplied, return
> a cell array containing the list of all files that have the same
> name in the path. If no files are found, return an empty cell array.
>
>
>
> **See also:** file\_in\_loadpath.

— Built-in Function: **tilde\_expand** (string)  
> Performs tilde expansion on string. If string begins with a
> tilde character, (~), all of the characters preceding the first
> slash (or all characters, if there is no slash) are treated as a
> possible user name, and the tilde and the following characters up to the
> slash are replaced by the home directory of the named user. If the
> tilde is followed immediately by a slash, the tilde is replaced by the
> home directory of the user running Octave. For example,
>
> ```
>           tilde_expand ("~joeuser/bin")
>                => "/home/joeuser/bin"
>           tilde_expand ("~/bin")
>                => "/home/jwe/bin"
> ```

— Built-in Function: [cname, status, msg] **canonicalize\_file\_name** (name)  
> Return the canonical name of file name.

— Function File: [status, msg, msgid] = **movefile** (f1, f2)  
> Move the file f1 to the new name f2. The name f1
> may contain globbing patterns. If f1 expands to multiple file
> names, f2 must be a directory.
>
> If successful, status is 1, with msg and msgid empty\n\
> character strings. Otherwise, status is 0, msg contains a\n\
> system-dependent error message, and msgid contains a unique\n\
> message identifier.\n\
>
>
>
> **See also:** glob.

— Function File: [status, msg, msgid] = **copyfile** (f1, f2, force)  
> Copy the file f1 to the new name f2. The name f1
> may contain globbing patterns. If f1 expands to multiple file
> names, f2 must be a directory. If force is given and equals
> the string "f" the copy operation will be forced.
>
> If successful, status is 1, with msg and msgid empty\n\
> character strings. Otherwise, status is 0, msg contains a\n\
> system-dependent error message, and msgid contains a unique\n\
> message identifier.\n\
>
>
>
> **See also:** glob, movefile.

— Function File: [dir, name, ext, ver] = **fileparts** (filename)  
> Return the directory, name, extension, and version components of
> filename.
>
>
>
> **See also:** fullfile.

— Built-in Function: **filesep** ()  
— Built-in Function: **filesep** ('all')  
> Return the system-dependent character used to separate directory names.
>
> If 'all' is given, the function return all valid file separators in
> the form of a string. The list of file separators is system-dependent.
> It is / (forward slash) under UNIX or Mac OS X, / and \ (forward and
> backward slashes) under Windows.
>
>
>
> **See also:** pathsep, dir, ls.

— Built-in Function: **filemarker** ()  
> Returns or sets the character used to separate filename from the
> the subfunction names contained within the file. This can be used in
> a generic manner to interact with subfunctions. For example
>
> ```
>           help (["myfunc", filemarker, "mysubfunc"])
> ```
>
> returns the help string associated with the sub-function `mysubfunc`
> of the function `myfunc`. Another use of `filemarker` is when
> debugging it allows easier placement of breakpoints within sub-functions.
> For example
>
> ```
>           dbstop (["myfunc", filemarker, "mysubfunc"])
> ```
>
> will set a breakpoint at the first line of the subfunction `mysubfunc`.

— Function File: filename = **fullfile** (dir1, dir2, ..., file)  
> Return a complete filename constructed from the given components.
>
>
>
> **See also:** fileparts.

— Function File: dir = **tempdir** ()  
> Return the name of the system's directory for temporary files.

— Function File: filename = **tempname** ()  
> This function is an alias for `tmpnam`.

— Built-in Function: **P\_tmpdir** ()  
> Return the default name of the directory for temporary files on
> this system. The name of this directory is system dependent.

— Built-in Function: **is\_absolute\_filename** (file)  
> Return true if file is an absolute filename.

— Built-in Function: **is\_rooted\_relative\_filename** (file)  
> Return true if file is a rooted-relative filename.

— Built-in Function: **make\_absolute\_filename** (file)  
> Return the full name of file, relative to the current directory.
